# *Helicobacter pylori* Infection, Virulence Genes' Distribution and Accompanying Clinical Outcomes: The West Africa Situation

**DOI:** 10.1155/2019/7312908

**Published:** 2019-12-10

**Authors:** Eric Gyamerah Ofori, Cynthia Ayefoumi Adinortey, Ansumana Sandy Bockarie, Foster Kyei, Emmanuel Ayitey Tagoe, Michael Buenor Adinortey

**Affiliations:** ^1^Department of Molecular Biology and Biotechnology, School of Biological Sciences, University of Cape Coast, Cape Coast, Ghana; ^2^Department of Biochemistry, School of Biological Sciences, University of Cape Coast, Cape Coast, Ghana; ^3^Department of Internal Medicine, School of Medical Sciences, University of Cape Coast, Cape Coast, Ghana; ^4^West Africa Centre for Cell Biology of Infectious Disease and Pathogens, Department of Biochemistry, Cell and Molecular Biology, University of Ghana, Legon, Ghana; ^5^Department of Medical Laboratory Sciences, School of Biomedical and Allied Health Sciences, University of Ghana, Ghana

## Abstract

Data on *Helicobacter pylori *(*H. pylori*) infection and virulence factors in countries across West Africa are scattered. This systematic review seeks to present an update on the status of *H. pylori *infection focusing on prevalence rate, distribution of virulent genes, and their link to clinical outcomes across countries in the western part of Africa. This information is expected to broaden the knowledge base of clinicians and researchers regarding *H. pylori* infection and associated virulence factors in West African countries. *Search Method.* A comprehensive search of the scientific literature in PubMed and ScienceDirect was conducted using the search terms including “*Helicobacter pylori* infection in West Africa”. Databases were sourced from January 1988 to December 2018. *Results.* Data on the incidence of *H. pylori* infection and related pathological factors were found for some countries, whereas others had no information on it. Smoking, alcohol, exposure to high levels of carcinogens and diet were reported to be involved in the pathogenesis of gastroduodenal diseases and gastric cancer. Besides the environmental factors and genetic characteristics, there are important characteristics of *H. pylori* such as the ability to infect, replicate, and persist in a host that have been associated with the pathogenesis of various gastroduodenal diseases. *Concluding Remarks.* This systematic search has provided information so far available on *H. pylori* virulence factors and clinical outcomes in West Africa. Accordingly, this piece has identified gaps in the body of knowledge highlighting the need for more studies to clarify the role of *H. pylori* virulence factors and associated clinical outcomes in the burden of this bacterial infection in West Africa, as data from these countries do not give the needed direct relation.

## 1. Introduction

*Helicobacter pylori *(*H. pylori*) are common microaerophilic bacteria known to obstinately inhabit the human stomach mucous layer, affecting about half the world's population [1–3]. The pathogen is known to be present in the mucous, on the surface of the stomach lining and its presence causes chronic inflammation, which remains a major cause of prolonged gastritis. Also, *H. pylori* has been identified to increase the risk of developing gastric adenocarcinoma [4]. Apart from the gastrointestinal tract (GIT) related diseases such as gastroesophageal reflux disease, gastric ulcer and duodenal ulcer, infection from *H. pylori *has also been linked to some other diseases such as iron-deficiency anemia [5, 6], immune thrombocytopenia (ITP), [5, 7] cardiovascular diseases, [8, 9], hepatobiliary diseases [10, 11], diabetes mellitus [12], allergies, and asthma [13] among others. Although Marshall and Warren [14] reported the first isolation of* H. pylori* in 1983, isolation of the organism is still not commonly done in West Africa with only few countries such as Senegal [15] and Ghana [16] having recorded their first successful isolation from gastric biopsy.

There is no certainty in the mode of transmission for *H. pylori* infection; however, various epidemiological studies have made several claims in this regard. The primary means of spread of the disease has been linked to transmission from one individual to another and usually higher when occurring within a family [17–19]. The spread from person-to-person has been identified to be the most likely and could be by oral-oral, gastro-oral or fecal-oral [20]. In this regard, the practice of good hygiene and improved living conditions becomes an essential factor in reducing the rate of transmission of the infection [21]. Infection occurs in children as well and an infected child maintains a strain, which has a genetic characteristic indistinguishable from that observed in their parents [22–24]. These characteristics remain unchanged upon any alteration in the environment in which they are found.

The prevalence of infection from *H. pylori* varies geographically with the developing world carrying the higher burden [25]. Infection in peptic ulcer diseases (PUDs) patients ranges from almost 25% in countries of the industrialized world, and is anticipated to be around 90% in underdeveloped countries [26, 27]. The prevalence across countries in the West African region is generally high with variations existing from country to country. The reportedly high prevalence observed in Africa (79.1%) and Asia (54.7%) as compared to lower prevalence found in other geographic locations such as Northern America (37.1%) and Oceania (24.4%) [28] have been found not to correlate with the rather low occurrence of gastric cancer [29, 30]. This situation has been described as the “Asian and African enigmas”. These so-called “enigmas” have been explained by several factors including host genetic and immune response, different tumor-inducing potential of explicit strains of *H. pylori* as well as environmental factors [29, 31]. Again, insufficient African population sampling obtained through endoscopy as well as poor access to health care has also been found to contribute to this so-called mystery. In this regard, a stronger and elaborate data on gastric ulcer in Africans and the prevalence of associated cancer have established that the low occurrence is not exactly so [32]. In contrast to the “Asian enigma”, a report by Irino et al. [33] shows that the incidence of gastric cancer (GC) is mainly high in Asian countries, a situation attributed to the high prevalence in infection from *H. pylori*.

It is imperative to mention that an individual that is infected with *H. pylori *faces a strenuous task of getting rid of the bacterium and hence disease eradication, a situation that is largely attributed to the ever-increasing antibiotic resistance [34]. In the near future, the problems associated with *H. pylori* eradication are feared to increase looking at the current increasing infection rates and the gastroduodenal pathological outcomes. It is estimated that about 15% of infected individuals have an increased tendency of developing peptic ulcer [35], while a rate of 1–3% are found to have a bigger propensity of developing gastric malignancy in their lifetime [36, 37]. With the changing epidemiology of infection from this organism, the pattern of other related diseases also keeps changing.

An extensive literature search has revealed that, the prevalence of *H. pylori* in countries across West Africa is generally high and it poses a serious health burden on health care systems. The overall impact varies from country to country. Several factors have been reported to contribute to this variation such as, host genetic factors, type of *H. pylori* virulence factors and sensitivity of the method of detection employed [38, 39]. There is inadequate information on the role of these factors in *H. pylori* infection rates and associated clinical outcomes across the countries in the Western area of Africa which makes tackling of the growing disease burden increasingly difficult. Assembling data on the type of virulent genes, factors involved in infection and associated clinical outcome is, therefore, important [40, 41]. This systematic review hence looks at available information in the West African zone on the prevalence of *H. pylori *infection, virulence factors and their relation to clinical outcomes.

## 2. Method of Literature Search

**Figure 1:**
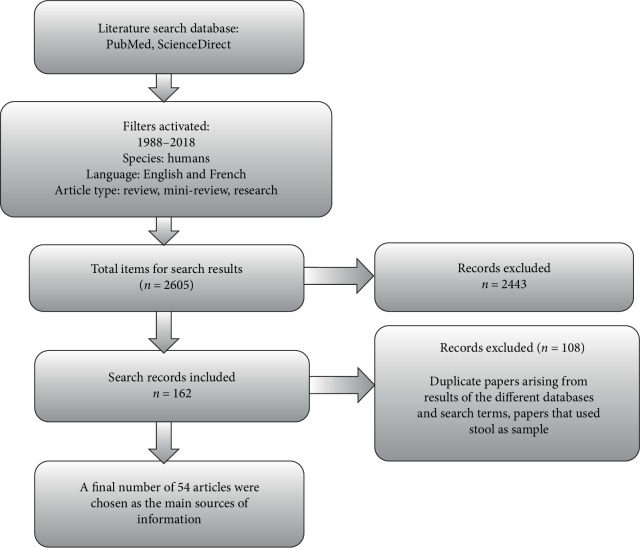
Search method employed to identify articles.

A comprehensive search of the scientific literature in PubMed and ScienceDirect was conducted using the search terms “*Helicobacter pylori* infection in West Africa”. The search was repeated with “West Africa” replaced with each of the following countries; Sierra Leone, Cape Verde, Ghana, Liberia, Benin, Senegal, Sao tome and Principle, Mali, Burkina Faso, Mauritania, Cote D'ivoire, Guinea Bissau, Niger, Guinea, The Gambia, and Togo. The key words “*Helicobacter pylori”* was replaced with “*H. pylori”* and the search was repeated. Some other keywords employed were “*Helicobacter pylori*”, “epidemiology”, “prevalence”, and “virulent factors”. The search protocol is shown in Figure 1.

The search in PubMed was done with the following activated filters; publication date from 1^st^ January 1988 to 31^st^ December 2018, language filter had English and French activated and the species selected was Human. Filters activated for the search in ScienceDirect database were the selection of the article types; Review articles, Research articles, and mini-reviews as well as the year range of 1988–2018. Duplicate searches were first removed; after which the abstracts of articles retrieved were reviewed for relevance before an attempt was made to retrieve the full paper. Selection of articles was based on the following considerations; (1) Study participants were West Africans. (2) Participants who showed up for endoscopy at a gastroenterology unit and diagnosed with *H. pylori* infection. (3) Studies investigating virulent factors; Vacuolating cytotoxin (*VacA*), Cytotoxin-associated gene A (*cagA*), Outer inflammatory protein (*OipA*), Duodenal ulcer promoting gene (*dupA*), Blood group antigen binding adhesin (*BabA*) and Induced by contact with epithelium (*IceA*) as a contributing factor to disease progression. (4) Obtained clinical features alongside the detection of virulence factors in order to compare how the presence of a factor correlates with disease outcome and manifestation.

Studies excluded were; (1) West Africans participants living outside the study region. (2) Case studies on an individual, and retrospective records review of patients, commentaries, editorials, and letters in response to published articles. (3) Detection of pathogen by stool antigen test. (4) Prevalence among a selected disease group e.g., AIDS and Diabetes patients. (6) Articles that covered prevalence in children alone.

## 3. Results

### 3.1. Prevalence of Helicobacter pylori Infection in West Africa

Infection from *H. pylori *is usually asymptomatic and affects nearly 50–75% of global population [42, 43], its prevalence varies between countries [44]. About 70% of people in developing countries with PUDs are estimated to be affected, though the proportions are slightly lower (25–50%) in the developed countries [42, 45]. The variation across countries is found to be due to an interplay of several factors including host immune response and genetic interaction as well as differences in the potential of specific strains of *H. pylori* to cause cancer. Other studies have attributed the variation in the geographic burden of this infection to age of individual, gender, ethnicity, and factors relating to the environment [29, 31, 46]. These factors are also involved in the variation of the rate of infection that is seen in some countries [3, 47, 48]. Also contributing to this variation in prevalence is socioeconomic status and a dependency on the rate of acquisition in the first 5 years of a person's life [48–51]. In the first 5 years of life, infection with *H. pylori *is reportedly lower in developed countries as compared to developing counterparts. This is possibly due to different degrees in hygiene related practices, which are supposedly better in the developed world at that stage of life. In Burkina Faso for instance, a higher rate of *H. pylori* infection was reported in the primary stages of life [52]. Colonization in early life by *H. pylori* has been found to predispose infected individuals to the progression of malnutrition and development faltering in The Gambia. These effects however, were found not to persist later in childhood [53]. Studies by Hunt et al. [25] and Kusters et al. [54] however, noted that, generally, infection in the developed countries remains substantially lower in children, but gradually increases with cumulative age. Prevalence in countries in the Middle East are also noted to mostly increase with age and rates are reportedly comparable to each other as well as to those occurring in the United States and Europe. Studies in countries such as Iraq, Iran, Israel, Libya, and Saudi Arabia have shown varied prevalence in various ages with rates in adults reported to be significantly higher than children [55]. Supporting the increasing prevalence in age is a report in The Gambia in which serological indication of *H. pylori* infection in 15% of infants less than 20 months was observed to increase to 46% in those aged 40–60 months [56]. The higher prevalence of *H. pylori* infection reported for this study is attributable to a birth cohort phenomenon.

In the West African region, a disease prevalence of 70–95% has been observed depending on the method used [44]. In Nigeria, a prevalence rate of 93.6% for *H. pylori* was found by serology, while 80.0% was estimated by histology [57]. The prevalence in Nigeria has, however, been found to generally range between 38.0% and 92% [58–62]. Surprisingly, an unusually low prevalence has been demonstrated in Mali where a rate of 21% was reported among persons with established gastric ulcer, 44% among the casually selected volunteers and 14% in those with gastrointestinal correlated illnesses. This low prevalence in the study population was attributed to the sensitivity of the detection kit [63] due to a possible *H. pylori* strain variability. Generally, the disease prevalence across countries in the West African area varies and there is no clear show of a steady overall increment or decrease with age. In Ghana, the reported prevalence rate is 75% [64–66] while in Senegal 62–97% has been reported [67–72]. A prevalence of 92% was seen in Cote D'Ivoire [73], while in Benin, about 56–72% has been reported [74, 75]. However, a lower prevalence of infection in adults compared to children has been reported in Burkina Faso contrary to earlier reports [52].

Notwithstanding the high rates of *H. pylori* infection globally, there is little evidence of what is being done to investigate or reduce this burden in some West African countries. Currently some countries including Mauritania, Guinea Bissau, Liberia, and Sierra Leone had no published research report that met the inclusion criteria, while others such as Benin registered no updated data in the research area in over a decade. With the high prevalence recorded for studied countries comparable to those of the Middle East and elsewhere in developing countries, overcrowded conditions are major contributing factors. These overcrowded conditions are noted to create closer contacts between mothers and their children as well as the sharing of same bed by siblings which are likely to be a leading reason for the increasing transmission rate and therefore higher infection rates [76].

#### 3.1.1. Influence of Host and *H. pylori* Genetic Factors on Infection

The distribution of *H. pylori* infection and the related pathology are mainly affected by host genetics and *H. pylori* virulence factors. In the progress of gastroduodenal diseases such as gastric cancer (GC), there seems to be an increase in the chances of development resulting from polymorphisms in several virulence genes. *H. pylori *genetic diversity is observed to be widespread and demonstrated in the disease outcome of various strains infection and pathogen interaction with their human host. Although not clearly described, the level of genetic multiplicity is considered as an element for adaptation of *H. pylori* in the stomach of the host and the clinical outcomes of the infection [77]. A high through-put sequencing revealed the diverse nature of *H. pylori* genome, [78] and the bacteria genes, variability was adopted to establish a vibrant phylogeographic distinction, a marker for human migrations [79, 80]. Human genetic polymorphisms also compliment geographical distribution of *H. pylori* and the clinical consequences. For example, persons carrying genetic variation in the proinflammatory interleukin-1-beta (IL-1b) and IL-1 receptor adversary genes are two to three times more probable to develop GC [81]. Similarly, polymorphic tumor necrotic factor (TNF)-α as well as IL-16 genes were found to favor GC development during *H. pylori* infection [82, 83].

#### 3.1.2. Environmental Factors in *Helicobacter pylori* Infection

Several factors have been reported to promote *H. pylori* infection and associated gastroduodenal diseases. Smoking, excessive alcohol intake, experience with high levels of carcinogens, and diet are proven to be significantly involved in the pathogenesis of gastroduodenal diseases including gastric cancer (GC) [84, 85]. Individuals with a higher intake of refined carbohydrates, pickled, salted, or smoked foods and dried fish and meat are at a higher risk of developing GC as compared to those who consume foods containing higher amounts of fiber, fresh vegetables, and fruits [85]. Antioxidant properties of micronutrients contained in foods with high amounts of fiber and fresh fruits and vegetables are capable of lowering the risk of GC development by exerting a positive effect on the mucosa of gastrointestinal tract. Research on the risk factors of infection in the West African region is very scanty. In Nigeria, while the eating of raw vegetables demonstrated no significance in relation to *H. pylori *infection, drinking of unpasteurized milk recorded a significant association with infection [86]. The study went ahead to demonstrate that the source of drinking water and *H. pylori *infection had no relationship. This is in contrast to another finding in Nigeria that reported a higher prevalence of the infection in individuals who sourced their drinking water from wells, streams, and ponds as compared to tap water [87, 88]. The possibility of fecal contamination was explained to have resulted in the unwholesomeness of water from the other sources besides the tap water.

Meanwhile, isolation of the organism from the intestinal tract of sheep, dogs and cats [89] exists and has been found to survive in a culture of fresh sheep-milk where survival is reported to extend to several days [90]. In a study, conducted in Burkina Faso, healthy individuals visiting a hospital for medical check-up were found to be infected with *H. pylori*, and they were found in shepherds or belonging to families of shepherds [52]. Therefore, study to clarify the role of sheep as zoonotic reservoir of *H. pylori *is highly recommended.

Tobacco usage has also been reported to be a dependent ulcerogenic risk factor for the development of gastroduodenal disorders including peptic ulcers and cancers of the GI tract [91]. Smoking acts indirectly in the promotion of gastroduodenal conditions by adversely affecting the mucosal protective mechanisms of the GI tract and also increasing the risk of *H. pylori* infection [92]. While the relation to the increase in infection may be due to antioxidant reduction or gastroduodenal immune system defense mechanism, the adverse effect on mucosal protective mechanisms can be attributed to an increase in the production of gastric acid coupled with a reduction in the production of bicarbonate [93]. Studies have shown that, smoking inhibits epithelial cell renewal resulting in the alteration of mucosal cell proliferation. This exposes the gastrointestinal tract to various aggressive factors leading to an improved likelihood of the induction of cell apoptosis during ulceration and ulcer healing [94, 95]. A US population-based study obtained within 1997–2003 has revealed that, the prevalence of ulcer disease in present and former smokers (11.43% and 11.52%) is almost twice that of never smokers (6.00%) [96]. Babaei et al. have identified smoking as a habit that is largely associated with PUD patients (85%) than gastritis nonPUD patients (14%). In a study conducted in the USA and Japan to ascertain a possible relationship between patients having functional dyspepsia and smoking, participants did not show any relation of smoking to the condition [97]. Elsewhere in Nigeria, a study has demonstrated the association where cigarette smoking was found to significantly increase the prevalence of *H. pylori* infection [88].

Alcohol has been recognized to play an active part in the development of gastroduodenal conditions and can do so even at lower concentrations. A lower concentration of alcohol is capable of inducing apoptosis and can increase the expression of alcohol dehydrogenase of the gastric adenocarcinoma cell lines [98]. The carcinogenic and harmful effect of alcohol consumption results from the increase in the expression of aldehyde dehydrogenase, cytochrome P_450_, alcohol dehydrogenase, and by inducing the production of reactive oxygen and nitrogen species [99–101]. In Nigeria, Mnena et al. [86] found no significant association between alcohol consumption and *H. pylori* infection and these findings are similar to those reported in Portugal, China and the United States [102–104]. Contrasting findings obtained from several studies continues to keep inconclusive the consequence of alcohol consumption in *H. pylori* infection. In a study where nondrinkers showed a significantly lower infection rate compared to drinkers, [105] a present study reports of a lower risk of infection in people who drink alcohol, compared to nondrinkers [106]. Effect of alcohol intake on *H. pylori* infection continues to remain a subject open for further investigation.

### 3.2. Pathogenesis of Infection and Clinical Outcomes

Besides environmental and genetic factors, there are other important characteristics of *H. pylori* such as the ability to enter, replicate, and persist in a host, which have been linked to the pathogenesis of gastroduodenal diseases [107, 108]. These factors come into play after the individual is exposed to the organism. When *H. pylori* first enters the host stomach, it is faced with the task of surviving the hostile acidic condition of gastric acid bath where pH could be as low as 1.5. The organism utilizes its urease activity to break down urea into ammonium ions and carbondioxide. Once in the stomach, *H. pylori* reside on the epithelial surface. Movement into the mucosal lining is flagella-mediated, after which specific interactions between host cell receptors and bacterial adhesins follows. By these interactions, the bacterium latches onto the gastric epithelium and survives a possible displacement by the forces generated from the passing of food down the digestive tract.

*H. pylori* possess several strain specific virulent genes aside the typical virulence factors such as urease (*UreA*) and outer membrane protein (OMP) [109]. These strain specific genes include Vacuolating cytotoxin gene A (*vacA*), Cytotoxin-associated gene A (*cagA*), Outer inflammatory protein (*OipA*), (Duodenal ulcer promoting gene (*dupA*), Blood group antigen binding adhesin (*BabA*) and Induced by contact with epithelium (*IceA*). Among these, *cagA* and *vacA *are considered most frequently reported entities associated with clinical outcomes of infection with *CagA* being the most studied. An added advantage of the close proximity of the bacteria to gastric epithelium besides the promotion of survival in the harsh pH environment allows for ease in scavenging for nutrients from host. The nutrients are made available when toxins such as cytotoxin-associated gene A (*cagA*) and the vacuolating cytotoxin gene A (*vacA*) from the bacteria, effectively harm the host tissues. The complement of strain-specific *H. pylori *toxin gene is a mark of its virulence and the damage triggered by these toxins may eventually result in the onset of clinical symptoms. For example, some studies have shown that *cagA* which is a powerful bacterial toxin, is particularly linked to acute gastritis, and gastric cancer development [110–114] while *vacA* is related to gastric adenocarcinoma [115, 116]. As such, the measure of virulence and explanation to the manifestation of clinical outcome of several cases of infection has primarily been linked to the capacity of the organism to produce any of these virulence factors.

The cytotoxic activities of *vacA* and *cagA* are reported to have a high correlation although their genes occupy different genomic regions [117]. An important relationship therefore exist between *vacA* and *cagA* [81] and *H. pylori *strains that express a combination of the alleles of *vacA* s1m1 and *cagA* represent the highest in virulence [118, 119]. Infections involving the expression of a combination of these alleles may lead to a serious epithelial damage [120, 121] which can lead to the development of severe gastric diseases as depicted in Table 1. Among the strains of *vacA*, studies have shown that infections involving subtype *vacA *s1m1 account for higher levels of inflammation in the gastric mucosa and increases the risk for carcinoma and gastric atrophy, as compared to the less virulent *vacA* s2m2 strains [83]. Nonetheless, the association of *vacA* subtypes with disease outcome is not always consistent as may be seen in reports from many countries [122, 123].

In the West African region, there is paucity of studies existing on the genotyping of although a few studies from countries such as Ghana, Senegal, Nigeria, and The Gambia show that majority of *H. pylori* strains have virulence factors [44]. Reports show inconsistent observations concerning the link of *vacA and cagA* with the sternness of disease as they occur at different geographic regions [124]. This means, there is no definite property of the bacterium in terms of virulence of the genotypes and that the selective inactivation of certain genes of virulence is an adaptation for host specificity [125]. Nonetheless, there is the likelihood of a global similarity in the mechanism responsible for differential antibiotic resistance.

#### 3.2.1. Cytotoxin-Associated Gene A

*CagA *is encoded on the cag pathogenicity island (*Cag*PAI). *Cag*PAI is a 40 kb region of chromosomal DNA that encodes nearly 31 genes forming a type IV secretion system. This system is noted for the injection of the oncoprotein, *cagA*, into mammalian cells [126] where it triggers cytokine production. Exploration of *cagA* gene is very popular among the *cag*PAI and constitutes the most documented virulence factor.

In line with a novel insertion sequence, *cagA* can be separated into two parts (regions) namely *cag* I and *cag* II [127] and depending on the repeat sequences of the 3′ region containing the Glu-Pro-Ile-Tyr-Ala (EPIYA) motifs, this gene can also be grouped as East-Asian-type or Western-type [127]. Although some specific EPIYA-like motifs (ESIYA and ESIYT) have been reported [128] in the C-terminal region of some isolated *CagA* strains elsewhere, the carcinogenic potential of *CagA* is greatly linked to its main polymorphic *EPIYA motif* variants, C and D [129]. *In vitro* studies have shown that the East-Asian-type *CagA* which contains EPIYA-D segments has an increased ability to induce morphological changes in epithelial cells and promote gastric cancer or peptic ulcer development than their Western-type counterpart that contains EPIYA-C segments [130]. In a different study however, EPIYA-C was identified to be an important factor in identifying patients with an increased risk of developing gastric cancer [131, 132]. The study of EPIYA motifs is poorly investigated in West Africa and, therefore, the link of the type that may be present to cause the disease cannot be clearly stated. Harrison et al. [61] identified that majority of the 111 study participants had a KDKGPE motif that was upstream an EPIYA-A motif. The study, however, did not investigate the function of this motif, and hence the link to pathology among Nigerians could not be clarified. Once *cagA* is introduced into the cells, it goes through phosphorylation by kinases of the host cell affecting cytoskeletal and tissue structure along with cell proliferation [83, 133]. *H. pylori* strains identified with this gene are capable of inducing apoptosis of the epithelial cells through the mitochondrial pathway and this compromises the barrier responsible for the protection of the epithelium against luminal acid and pepsin [134].

The *cagA *gene is associated with greater outcomes of inflammation and is involved in some severe forms of gastrointestinal diseases such as peptic ulcer and GC [111–113]. Elsewhere in the more developed world, reports have shown that persons infected with *H. pylori* that are positive for *cagA* strains are at a greater risk of developing peptic ulcer or GC than those that are *cagA*-negative [135]. Meanwhile, in East Asia, most strains of *H. pylori* have the *cagA* gene regardless of the disease [136].

Among the few countries in the West African region to sequence the *H. pylori* gene, there have been reported associations between *cagA* and the prevalence of the various diseases. For instance, in a cross-sectional study of 113 *H. pylori* positive Ghanaian population with dyspepsia, the prevalence of the organism harboring the *cagA *virulence factor was found to be 74.8% and a persistent association existed between *cagA*-(hydrophilic region) and duodenal ulcer (Table 1) [66]. A high prevalence 90–97% has also been demonstrated in Nigeria [61, 137, 138]. A study by Smith et al., found the presence of *cagA* infection in 91% of patients in Nigeria with nonulcer dyspepsia and 95% of them had duodenal ulcer. The study, however, concluded that no association existed between the studied genotypes and duodenal ulcer disease in that particular population [139]. These reported observations on prevalence are consistent with studies in India (96% among duodenal ulcer patients), [140] Gauteng (87% among asymptomatic children age between 6 and 15 years), [141] Alaskans-US (85%), [142] China (89.3% in patients with upper gastrointestinal diseases), and Taiwan (83% in isolates from patients with chronic gastritis and peptic ulcer) [143]. Another report in Nigeria by Mnena et al. [86], however, identified only 29% out of 22 *H. pylori* positive patients to have the *cagA* gene. The rather low prevalence of *cagA * in that population explains the low rate of recurrence of severe gastrointestinal disorders among the studied patients. Elsewhere in Senegal, a total of 117 *H. pylori* culture-positive patients yielded 73.3% of the *cagA* gene and this was observed to be strongly related to GC. Breurec et al. [70] also found 73.3% of isolates from Senegalese patients to be positive for *cagA* gene which was also associated with GC. These findings are synonymous to findings reported elsewhere on the African continent [78, 144]. Similar results also exist for bacterial strains with *cagA* in countries such as Iraq, Iran, and Turkey with a reported prevalence of 71%, 76%, and 78%, respectively. The presence of *cagA* identified was found to have a significant association with the incidence of peptic ulcer disease in Turkey and Iraq but not in Iran [55, 145]. Also, in The Gambia, similar results were obtained for the proportion of samples that were *cagA* positive using DNA from biopsies and culture. A prevalence of 58.3% and 61.7% were recorded for DNA from biopsies and culture, respectively [61, 146, 147]. Generally, 61.2% of these Gambian patients were found to have the *cagA* gene only, while 17.4% were positive for the *cag* empty site only. A rate of 19% was also found to be positive for both. The study, however, did not state clearly the relation of the detected gene to the pathology of the disease.

#### 3.2.2. Vacuolating Cytotoxin Gene A (vacA)

The *vacA* is a 140-kDa polypeptide that is secreted from the bacteria and delivered in an active form to host cells, where it exerts its activity [148]. All *H. pylori* strains contain the gene that encodes *vacA* although not all are fully cytotoxic [135, 149]. The *vacA * gene has three regions of genetic allelic diversity namely; intermediate (i1 and i2), the signal (s1 and s2), and the middle regions (m1 and m2) which determines the difference in vacuolating abilities [115, 121]. Damage to epithelial cells by the *vacA* gene is achieved by inducing the formation of vacuoles. The degree of cytotoxic activity of the toxin varies from strain to strain [83, 150] with the highest vacuolating activity occurring in s1/m1 genotypes. Activity is intermediate in s1/m2 genotypes and absent in s2/m2 genotypes [135]. Among the types, the s1/i1/m1 *vacA* is repeatedly associated with genopositive *cagA* [151] and neither of the virulence indicators is considered a self-regulating influence for the outcome of the disease [54]. The risk of severe clinical outcome is therefore greater when several virulence markers exist. Apart from the impact of the differences in *vacA * toxicity among strains, the expression of *vacA * during *H. pylori* infection varies widely and is associated with the degree of inflammation and presence of atrophy [152]. This implies that the risk of development of disease by an *H. pylori* infected person is not only reliance on the type of *vacA * of the infecting strain but also on the level of expression of the gene [152]. The study by Sinnet et al. and Amilon et al. [153] has shown that *vacA* expression level and gastric inflammation is associated with polymorphism at nucleotide +28 with the *vacA* 5′ untranslated region of the transcript. Such effects on *vacA* transcript levels are important in their provision of possible additional risk markers for determining patients at a higher risk of developing severe duodenal or gastric diseases [152].

The presence of *vacA* is therefore very vital in the disease outcome of *H. pylori* infection and hence its identification in infected individuals could be very helpful in prediction, diagnosis, and subsequent treatment approach of infection. In several of the research performed on *H. pylori* in the studied countries, only a few delve into the virulence factors. Among Ghanaian patients presenting with dyspepsia, the prevalence of *vacAs1m1 *was found to be 25.2% and that of *vacAs1m2* was 8.2%. Majority of patients who recorded a positive result for the presence of *vacAs1* were also positive for *cagA* [66]. Infection with *vacA s1m1H. pylori* genotypes have been found to be associated with an increased risk of duodenal ulcer disease and this was evident in the study by Archampong et al. [142]. The association identified in this study was consistent with other studies [46, 154].

In Nigeria, Smith et al. detected 98% of *vacA*s1 in 40 *H. pylori* strains [139] while Harrison et al. found 92.8% detectable *vacA* levels [61] similar to the 90.6% obtained in South Africa [141]. In the study by Harrison et al., a bulk of the isolates harbored the *vacA* s1, m2 genotype, followed by the s1, m1 genotype. There was no detection of the *vacA* s2 genotype, consistent with the outcome of a study by Wei et al. [155]. Harrison et al. also detected no *vacA *m1 and results obtained for the two patient groups being those presenting with duodenal ulcer disease and nonulcer dyspepsia showed no significant difference. Mnena et al. [86] found the following among Nigerian dyspeptic patients; For the *vacA* genotypes, the s1c/m2 genotype formed 79% of the *H. pylori* infections while 8% was found for s1b/m2 genotype similar to findings in China in which 69.5% and 2.5% were detected for s1cm2 and s1bm2, respectively [155]. Occurring at 4% each were three different genotypes; s1c/m1, s1c/m1/m2, and s1c/s2/m2. The most prevalent (83%) among the various genotypes was the moderate virulence type of s1m2. The most virulent (s1m1) and least virulent (s2m2) genotypes were found to be 8% and 4% respectively. In the Gambia, the more toxigenic *VacA* s1 and m1 gene were demonstrated in 76.9% and 45.5% of subjects, respectively [146, 147], although the relation to clinical outcome was not clearly stated. *vacAs*2 and *vacAm*2 were, however, found to be at a rate of 19% and 29.8% respectively. Among Senegalese patients, there was a detection of high-vacuolization isotypes in which 57.1% were s1im1 subtype, while 21.9% had the s1im2 subtype. The s1 *vacA* allele were found to be associated with GC [70].

#### 3.2.3. Induced by Contact with Epithelium Gene A (IceA)

*IceA *has two central allelic variants namely, *iceA1* and *iceA2* [156, 157] and the relationship between them and clinical outcome is quite controversial. While some studies have proven that *iceA1*/*iceA2* may be directly involved in diseases of the gastrointestinal system, [158, 159] others have demonstrated contrary findings [115, 116]. *IceA1* is, however, upregulated upon the contact of *H. pylori* with the gastric epithelium and has been found to be a probable marker for peptic ulcer disease while *IceA2 *is not considered a molecular marker of more virulent *H. pylori* strains [160, 161]. In a study by Smith et al. [139] conducted in Nigeria, all *H. pylori* isolates contained the* iceA *gene. In total, a considerably higher rate of 90.2% (37 isolates) were positive for *iceA*1 out of which 94.7% (18 isolates) were obtained from duodenal ulcer patients while 86.4% (19 isolates) were from nonulcer dyspepsia patients. Only one isolate from a nonulcer dyspepsia patient yielded both *iceA1* and* iceA2* in the PCR product. Babaei et al. [91] have confirmed that there is a strong relationship between duodenal ulcer and the genotypes of *iceA1*(+)/*iceA2*(−) [91]. The study identified a 61% *iceA1*(+)*/iceA2*(−) prevalence in peptic ulcer patients and 17.6% prevalence of this genotype for *H. pylori* infected nonpeptic ulcer disease patients. Wei et al. [155] have also recorded a gastric cancer associated *iceA1* prevalence of 70.1% which were stated to be consistent with findings from Korea, China, Tunisia, and Thailand.

## 4. Concluding Remarks and Further Studies

The understanding of *H. pylori *infection and disease progression has improved over the past few years in countries of West Africa although prevalence of infection is generally high. Available data indicate that studies on virulence factors are inadequate although few countries with research data on it studied factors such as *cagA* and *vacA* and observed a high prevalence. While information in literature has demonstrated the role of other factors such as *BabA2*, *OipA*, *dupA IceA*, the genes that encode for glycosyl transferases and the allelic variants of some of the studied factors on the pathology of *H. pylori* infection, these areas are insufficiently dealt with in studies across countries of the Western part of the African continent. Expounding the roles of *H*. *pylori* virulence factors in pathogenesis and clinical outcomes would greatly benefit vaccine and alternative drug therapy development.

## Figures and Tables

**Table 1:** *H. pylori *virulence factors and related clinical outcome in some West Africa countries.

Country	*H. pylori* genotypes	Clinical outcomes	Reference
Ghana	*vacAs1m1*	Increased risk of duodenal ulcer disease	[66]
	*vacAs1m2*	Duodenal ulcer (reduced risk as compared to *vacAs1m1*)	
	*cagA*13-(hydrophilic region)	Duodenal ulcer	
	*cagA*24-(region of internal duplication)	Erosive gastritis	
Nigeria	*iceA1*	Normal pathology	[61]
	*iceA2*	Normal pathology	
	*vacAs1m1*	Normal pathology	
	*vacAs1m2*	Normal pathology	
	*vacAs2m1*	Normal pathology	
	*vacAs2m2*	Normal pathology	
	*cagA*	Normal pathology	
Senegal	*cagA*	Gastric cancer	[70]
	*vacAs1*	Gastric cancer	
	*vacAm1*	Not associated with an enhanced risk of Gastric cancer	
Gambia	*vacAs1*	Not correlated with disease outcomes	[146]
	*vacAs2*		
	*vacAm1*		
	*vacAm2*		
	*cagA*		
	*iceA1*		
	*iceA2*		
